# Interstitial Brachytherapy for Early Oral Cavity Squamous Cell Carcinoma: Long-Term Treatment Outcomes From a Single Tertiary Oncology Center

**DOI:** 10.7759/cureus.95065

**Published:** 2025-10-21

**Authors:** Carol Wong, Kai Cheong Roger Ngan, Chung Hang James Chow

**Affiliations:** 1 Department of Clinical Oncology, Queen Elizabeth Hospital, Hong Kong, HKG; 2 Department of Clinical Oncology, Gleneagles Hospital Hong Kong, Hong Kong, HKG

**Keywords:** head and neck brachytherapy, head and neck cancer radiotherapy, interstitial brachytherapy, oral cavity cancer, oral cavity squamous cell carcinoma

## Abstract

Purpose/objective

This study aimed to evaluate the long-term efficacy of interstitial brachytherapy for the treatment of early oral cavity squamous cell carcinoma (OCSCC).

Materials and methods

This retrospective review included consecutive patients with early OCSCC (cT1-2N0M0) who underwent primary radiotherapy exclusively by interstitial brachytherapy between January 2000 and December 2020. The primary outcome was local recurrence-free survival (LRFS); secondary outcomes included regional recurrence-free survival (RRFS), disease-free survival (DFS), disease-specific survival (DSS), overall survival (OS), and treatment-related complications. Incidence of late treatment-related complications is reported.

Results

Thirty-eight patients with early OCSCC underwent interstitial brachytherapy during the study period, of which 20 (52.6%) underwent high dose rate (HDR) brachytherapy with a median dose of 55 Gy and 18 (47.4%) underwent low dose rate (LDR) brachytherapy with a median dose of 66.5 Gy. Elective neck dissection was performed in 33 (86.8%) patients. At a median follow-up time of 11.8 years, the three-year and five-year LRFS were 86.1% and 76.6%, respectively. RRFS was 80.2% at three years and 77.1% at five years; DFS was 73% at three years and 62.2% at five years; DSS was 88.6% at three years and at five years; OS was 78.4% at three years and 75.7% at five years. Local recurrence occurred in 10 (26.3%) patients, with six (60%) salvaged by surgery. Regional recurrence occurred in eight (21.1%) patients, and three (37.5%) were salvaged by neck dissection. Treatment-related complications included persistent tongue ulcer in three (7.9%) patients and osteoradionecrosis of the jaw in four (10.5%) patients.

Conclusion

Interstitial brachytherapy for early OCSCC yielded acceptable long-term local control and safety data. While radical surgery continues to be the standard of care for OCSCC, interstitial brachytherapy remains an effective and safe alternative for selected patients with early-stage disease.

## Introduction

For early-stage oral cavity squamous cell carcinoma (OCSCC), surgery is considered the standard of care. Definitive radiotherapy, including interstitial brachytherapy, is an alternative in selected patients [1,2] who prefer organ preservation and wish to avoid the potential long-term surgical sequelae.

For definitive radiotherapy, both external beam radiotherapy and brachytherapy are options that can be considered. Brachytherapy provides the advantage of delivering a high radiation dose to the target volume with rapid dose fall-off [3]. Compared to intensity modulated radiotherapy (IMRT), brachytherapy can achieve a comparable level of conformity in patients with cT1-2N0 OCSCC while maintaining a considerably lower dose to the adjacent organs at risk [3], thereby potentially reducing the risks of xerostomia or trismus [4].

Surgery for oral cavity cancer, with or without adjuvant radiotherapy, may result in various long-term side effects, such as altered speech and swallowing, pain, and dysgeusia; these side effects are associated with a reduced quality of life in long-term survivors [5]. The treatment objective for oral cavity cancer includes both eradication of the tumor and, when feasible, preservation of function [6]. Brachytherapy provides an option for organ preservation and has the advantage of better cosmetic and functional outcomes [7]. Most patients treated with brachytherapy were able to regain normal function and experienced a favorable long-term quality of life [5]. Previous studies that evaluated the quality of life and functional outcomes by the European Organization for Research and Treatment of Cancer (EORTC) Quality of Life Questionnaire Core 30 (QLQ-C30) showed that patients with OCSCC who were treated by high dose rate (HDR) or low dose rate (LDR) brachytherapy could at least return to their baseline QLQ-C30 score one year after completion of treatment [8,9], but those who were treated by surgery experienced decline in certain functional domains of QLQ-C30 one year after completion of treatment [10].

Previous studies on the management of OCSCC by interstitial brachytherapy were heterogeneous. A considerable proportion of studies included individuals treated exclusively with brachytherapy, as well as those who received combination therapy with external beam radiotherapy and/or concurrent chemotherapy. This variability in therapeutic approaches and the relatively limited sample sizes may complicate the interpretation of treatment outcomes. This study, therefore, focused on patients with early OCSCC who underwent exclusive interstitial brachytherapy to the primary tumor, with the objective of analyzing the long-term outcomes, patterns of failure, and prognostic factors associated with disease control.

This article was previously presented as a meeting abstract at the ESTRO 2025 on May 5, 2025.

## Materials and methods

Patient characteristics

This is a single-institution retrospective study. Consecutive patients with histologically proven OCSCC who underwent primary interstitial brachytherapy between January 2000 and December 2020 at the Department of Clinical Oncology, Queen Elizabeth Hospital, were included. All patients had early-stage disease (cT1-2N0M0, staged by the American Joint Committee on Cancer (AJCC) fifth, sixth, or seventh edition, depending on the year of diagnosis) with no history of prior radiotherapy to the head and neck. Details of patient characteristics are presented in Table 1.

**Table 1:** Patient demographics and tumor characteristics ECOG: Eastern Cooperative Oncology Group

Characteristics	Number	%
Age (median, range)	66 (25-87)	
Sex		
Male	14	36.8
Female	24	63.2
ECOG performance status		
0	3	7.9
1	31	81.9
2	1	2.6
Unknown	3	7.9
Smoking status		
Non-smoker	25	65.8
Ex-smoker	4	10.5
Smoker	8	21.1
Unknown	1	2.6
Site of primary tumor		
Tongue	35	92.1
Buccal mucosa	3	7.9
Stage		
cT1N0	21	55.3
cT2N0	17	44.7
Tumor thickness (median, range) (cm)	0.7 (0.2-2.0)	
Tumor size (median, range) (cm)	2.0 (0.3-3.0)	

Treatment details

Prior to treatment, all patients were evaluated at the multidisciplinary meeting in the presence of surgeons, clinical oncologists, and diagnostic radiologists. For patients with early-stage OCSCC, treatment options with surgery or brachytherapy were offered. For patients who opted not for surgery, they would receive radical radiotherapy by interstitial brachytherapy.

All patients in this study were treated exclusively with interstitial brachytherapy without external beam radiotherapy as their primary treatment. Patients were treated by low-dose rate (LDR) brachytherapy before 2008 and by high-dose rate (HDR) technique thereafter. Both HDR and LDR used iridium-192 as the radiation source.

Implantation of brachytherapy catheters was performed by the guide-gutter technique or the plastic loop method with patients under general anesthesia. For the guide-gutter technique, the guide-gutters were inserted onto the tongue parallel and equidistant from each other; the insertion usually started with the second-most posterior or the middle guide-gutter. For the plastic loop method, catheters were inserted via the submental skin with the exit site on the tongue dorsum. Rules of the Paris system were followed for both techniques. After the insertion of guide-gutters or plastic loops and confirmation of their positions by orthogonal radiographs, the radiation source could be loaded into the implanted devices. Customized mandibular spacers, made from wax-embedded lead shields, were utilized to protect the mandible from the radiation exposure. All patients underwent dental assessment before treatment. Antiseptic mouthwash and a short course of steroids would be prescribed to maintain oral hygiene and reduce tumor edema, respectively. Further details on the techniques of interstitial implants performed in Queen Elizabeth Hospital were described in a previous publication [11]. The radiation dose delivered by HDR technique was 55 Gy (5.5 Gy per fraction) or 52 Gy (5.2 Gy per fraction) with a median of 55 Gy. HDR brachytherapy was administered as two fractions per day with a minimum of six hours in between. The radiation dose given by LDR technique ranged from 60 to 70 Gy with a median dose of 66.5 Gy.

Elective neck dissection was performed at the discretion of the treating physician. Adjuvant external beam radiotherapy to the neck, administered at doses of 54-60 Gy in 30 daily fractions, was given in cases of pathologically involved lymph nodes.

All patients were monitored clinically by physical examination upon follow-up. Additional investigations, such as imaging or biopsy, were arranged with respect to clinical findings.

Statistical analysis

The primary outcome was local recurrence-free survival (LRFS); secondary outcomes included regional recurrence-free survival (RRFS), disease-free survival (DFS), disease-specific survival (DSS), overall survival (OS), and treatment-related complications. LRFS was defined as the time from the date of commencement of brachytherapy to the first event of local recurrence in the oral cavity or death of any cause. RRFS was defined as the time from the date of commencement of brachytherapy to the first event of recurrence in the neck lymph node or death of any cause. DFS was defined as the time from the date of commencement of brachytherapy to the first event of recurrence or death of any cause. DSS was defined as the time from the date of commencement of brachytherapy to death due to oral cavity cancer. OS was defined as the time from the date of commencement of brachytherapy to death from any cause.

Survival endpoints were estimated using the Kaplan-Meier method. Prognostic factors were analyzed using the Cox regression and Fisher’s exact test. A p-value of less than 0.05 was considered to indicate a statistically significant difference. All analyses were performed using the SPSS statistical software version 30.0 (IBM Corp., Armonk, NY).

## Results

Treatment details

Twenty (52.6%) patients underwent HDR brachytherapy, and 18 (47.4%) patients underwent LDR brachytherapy. Of the 38 patients, 33 (86.8%) underwent elective neck dissection following primary interstitial brachytherapy. For the five patients who did not undergo elective neck dissection, three patients declined surgery and two patients were not offered elective neck dissection due to a thin tumor with a tumor thickness within 2 mm. There were no significant complications resulting from elective neck dissection.

For patients who underwent elective neck dissection, of the 33 patients, nine (27.3%) were found to have lymph node involvement by pathological examination. Details regarding elective neck dissection are presented in Table 2.

**Table 2:** Evaluation of the neck Table 2 shows the details of elective neck dissection, including the summary of pathological results and time from last fraction of brachytherapy to surgery.

Characteristics	Number	%
Number of lymph nodes dissected (median, range)	20 (5-56)	
Time (in days) between last fraction of brachytherapy to surgery (median, range)	40 (22-60)	
Pathological nodal status		
pN0	24	71.7
pN1	2	6.1
pN2	4	12.2
pN3	3	9.1
Number of involved lymph node among patients with pathological nodal involvement (median, range)	2 (1-3)	
Presence of extranodal extension among patients with pathological nodal involvement	5	15.1

Adjuvant radiotherapy to the neck was given to six (66.7%) of the patients who had node-positive disease on neck dissection. For the three patients who did not receive adjuvant radiotherapy, two of them declined and one of them had disease relapse before the start of adjuvant treatment. The median prescription dose of adjuvant external beam radiotherapy to the neck was 60 Gy in 30 daily fractions over six weeks.

Survival

At a median follow-up time of 11.8 years, 20 (52.6%) patients died.

The LRFS was 86.1% at three years and 76.6% at both five years and 10 years. The LRFS in the total population and by stage is shown in Table 3.

**Table 3:** Local recurrence-free survival Table 3 shows the three-year, five-year, and 10-year local recurrence-free survival in the total population and by stage. LRFS: local recurrence-free survival

	3-year LRFS	5-year LRFS	10-year LRFS
Total population	86.1%	76.6%	76.6%
Stage I	95.0%	84.4%	84.4%
Stage II	74.5%	66.2%	66.2%

Patients with stage II disease showed a numerically lower LRFS than those with stage I, with a hazard ratio (HR) of 3.873 (95% confidence interval (CI): 0.997-15.048, p=0.051). Other tested factors did not significantly impact the LRFS on univariate analysis, with details shown in Table 4.

**Table 4:** Evaluation of prognostic factors of LRFS, RRFS, DSS, and OS Table 4 shows the evaluation of baseline demographic factors, disease factors, and treatment factors and their relationship to LRFS, RRFS, DSS, and OS by univariate analysis. LRFS: local recurrence-free survival, RRFS: regional recurrence-free survival, DSS: disease-specific survival, OS: overall survival, CI: confidence interval

Factor	LRFS		RRFS		DSS		OS	
	Hazard ratio (95% CI)	p-value	Hazard ratio (95% CI)	p-value	Hazard ratio (95% CI)	p-value	Hazard ratio (95% CI)	p-value
Age	0.958 (0.921-0.996)	0.032	1.001 (0.955-1.050)	0.956	0.972 (0.929-1.017)	0.219	1.021 (0.987-1.056)	0.235
Gender	0.591 (0.164-2.129)	0.421	0.439 (0.109-1.765)	0.246	0.556 (0.124-2.501)	0.444	0.382 (0.158-0.925)	0.033
Performance status	0.682 (0.114-4.083)	0.675	0.475 (0.071-3.176)	0.442	0.558 (0.084-3.720)	0.547	2.269 (0.465-11.082)	0.311
Smoking status	2.040 (0.569-7.309)	0.273	5.100 (1.204-21.614)	0.027	2.328 (0.518-10.462)	0.270	2.585 (1.066-6.271)	0.036
Site of tumor	0.044 (0.000-1334)	0.553	0.044 (0.000-5.144E3)	0.600	0.043 (0.000-5.562E3)	0.601	1.676 (0.377-7.446)	0.497
Stage of tumor	3.873 (0.997-15.048)	0.051	2.556 (0.607-10.759)	0.201	4.106 (0.792-21.281)	0.093	1.368 (0.565-3.312)	0.488
Tumor size	1.557 (0.491-4.942)	0.452	2.401 (0.665-8.664)	0.181	1.653 (0.421-6.489)	0.471	1.199 (0.561-2.561)	0.639
Tumor thickness	0.148 (0.009-2.401)	0.179	1.032 (0.229-4.649)	0.967	0.962 (0.140-6.613)	0.969	0.811 (0.305-2.157)	0.675
Type of brachytherapy (HDR or LDR)	1.079 (0.312-3.735)	0.904	0.650 (0.155-2.723)	0.556	1.479 (0.330-6.622)	0.609	0.996 (0.394-2.517)	0.993
Time from diagnosis to first brachytherapy fraction	0.932 (0.502-1.730)	0.823	1.067 (0.549-2.074)	0.848	1.005 (0.472-2.140)	0.989	0.938 (0.586-1.502)	0.789
Number of neck nodes in neck dissection	-	-	0.949 (0.867-1.039)	0.255	1.036 (0.969-1.107)	0.300	0.996 (0.948-1.047)	0.884
Pathological neck staging	-	-	0.773 (0.267-2.234)	0.634	1.583 (0.761-3.291)	0.219	1.732 (1.088-2.756)	0.021
Number of positive lymph nodes	-	-	0.496 (0.106-2.329)	0.374	1.186 (0.577-2.437)	0.642	1.356 (0.875-2.104)	0.173
Presence of extranodal extension	-	-	90.416 (0.000-1.172E9)	0.590	77.071 (0.001-7.152E6)	0.457	130.866 (0.068-2.521E5)	0.207
Whether a neck dissection was performed	-	-	0.497 (0.100-2.464)	0.497	1.019 (0.123-8.468)	0.986	0.653 (0.218-1.958)	0.447
Time from last brachytherapy fraction to neck dissection	-	-	0.907 (0.814-1.011)	0.079	0.979 (0.888-1.080)	0.673	1.008 (0.948-1.073)	0.791
Adjuvant radiotherapy in pathological node-positive patients	-	-	34.176 (0-3.12E9)	0.706	32.903 (0-1.91E7)	0.606	0.580 (0.096-3.522)	0.554

**Figure 1:**
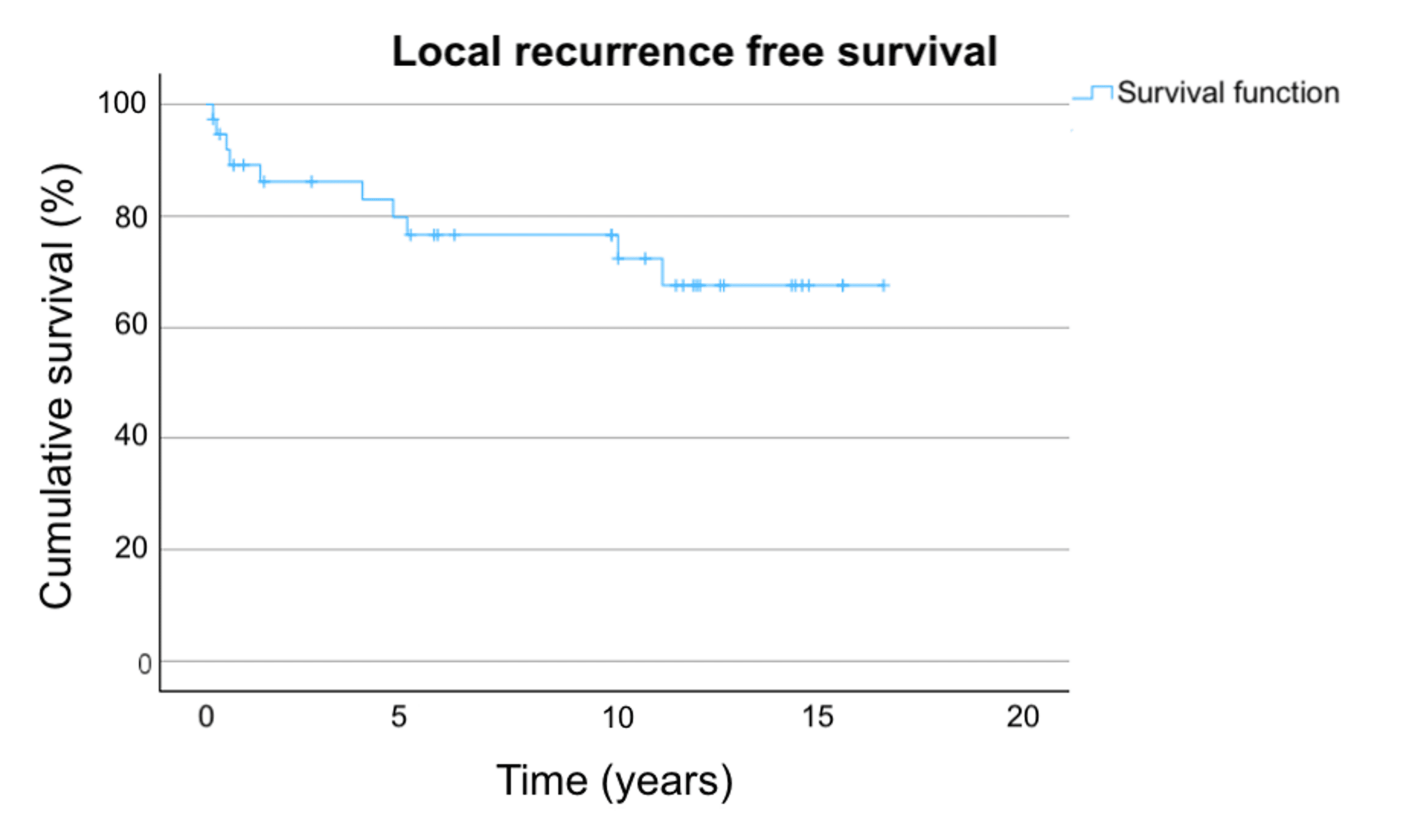
LRFS in overall population Figure 1 shows the LRFS in the overall population. The three-year, five-year, and 10-year LRFS are 86.1%, 76.6%, and 76.6%, respectively. LRFS: local recurrence-free survival

**Figure 2:**
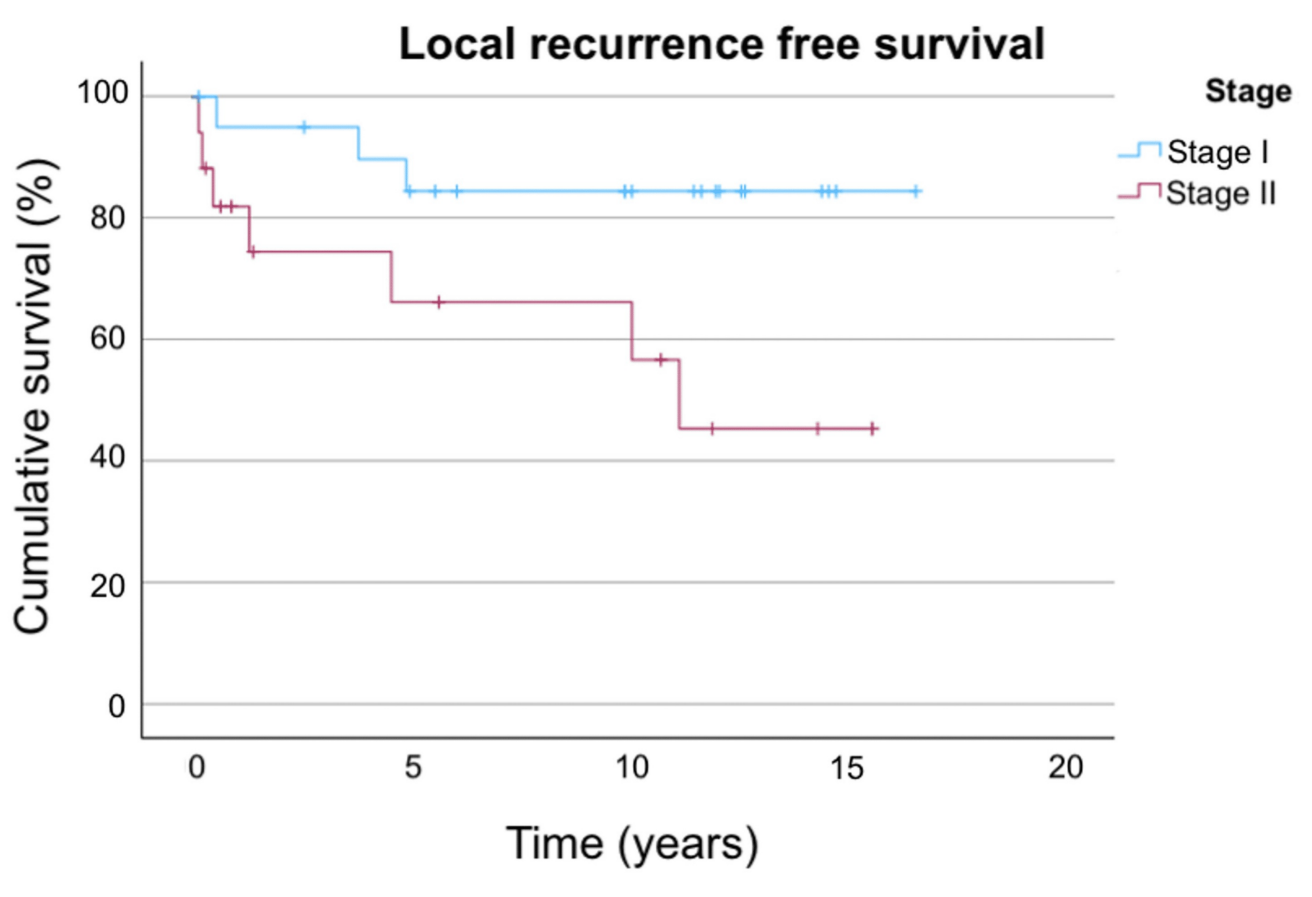
LRFS stratified by stage Figure 2 shows the LRFS stratified by stage. For stage I disease, the three-year, five-year, and 10-year LRFS are 95.0%, 84.4%, and 84.4%, respectively. For stage II disease, the three-year, five-year, and 10-year LRFS are 74.5%, 66.2%, and 66.2%, respectively. LRFS: local recurrence-free survival

The LRFS in the overall population and stratified by stage are shown in Figure 1 and Figure 2, respectively.

The RRFS was 80.2% at three years and 77.1% at both five years and 10 years. Smoking was a significant factor that negatively impacted the RRFS; patients who had a smoking history (current smoker or ex-smoker) have a hazard ratio of 5.10 (95% CI: 1.20-21.61, p=0.027) compared to non-smokers. Time from last fraction of brachytherapy to date of neck dissection showed a trend toward statistical significance (HR: 0.907, 95% CI: 0.814-1.011, p=0.079). Other tested factors did not significantly impact the RRFS on univariate analysis, with details shown in Table 4.

**Figure 3:**
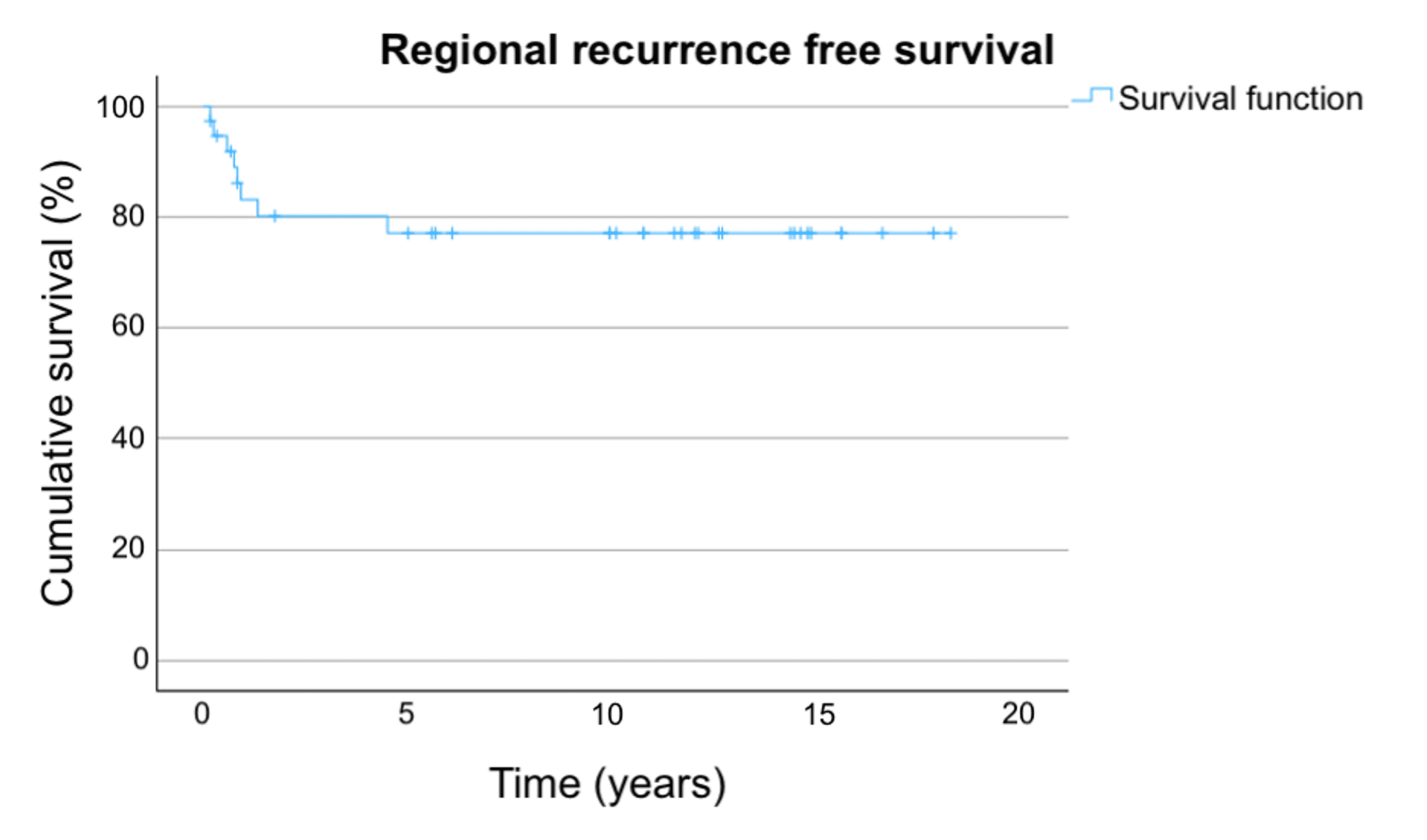
RRFS in overall population Figure 3 shows the RRFS in the overall population. The three-year, five-year, and 10-year RRFS are 80.2%, 77.1%, and 77.1%, respectively. RRFS: regional recurrence-free survival

**Figure 4:**
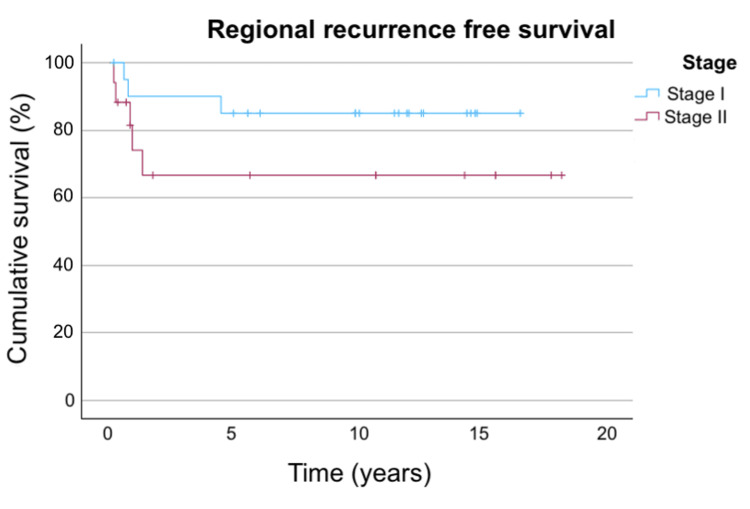
RRFS stratified by stage Figure 4 shows the RRFS stratified by stage. For stage I disease, the three-year, five-year, and 10-year RRFS are 90%, 85%, and 85%, respectively. For stage II disease, the three-year, five-year, and 10-year RRFS are all 66.6%. RRFS: regional recurrence-free survival

**Figure 5:**
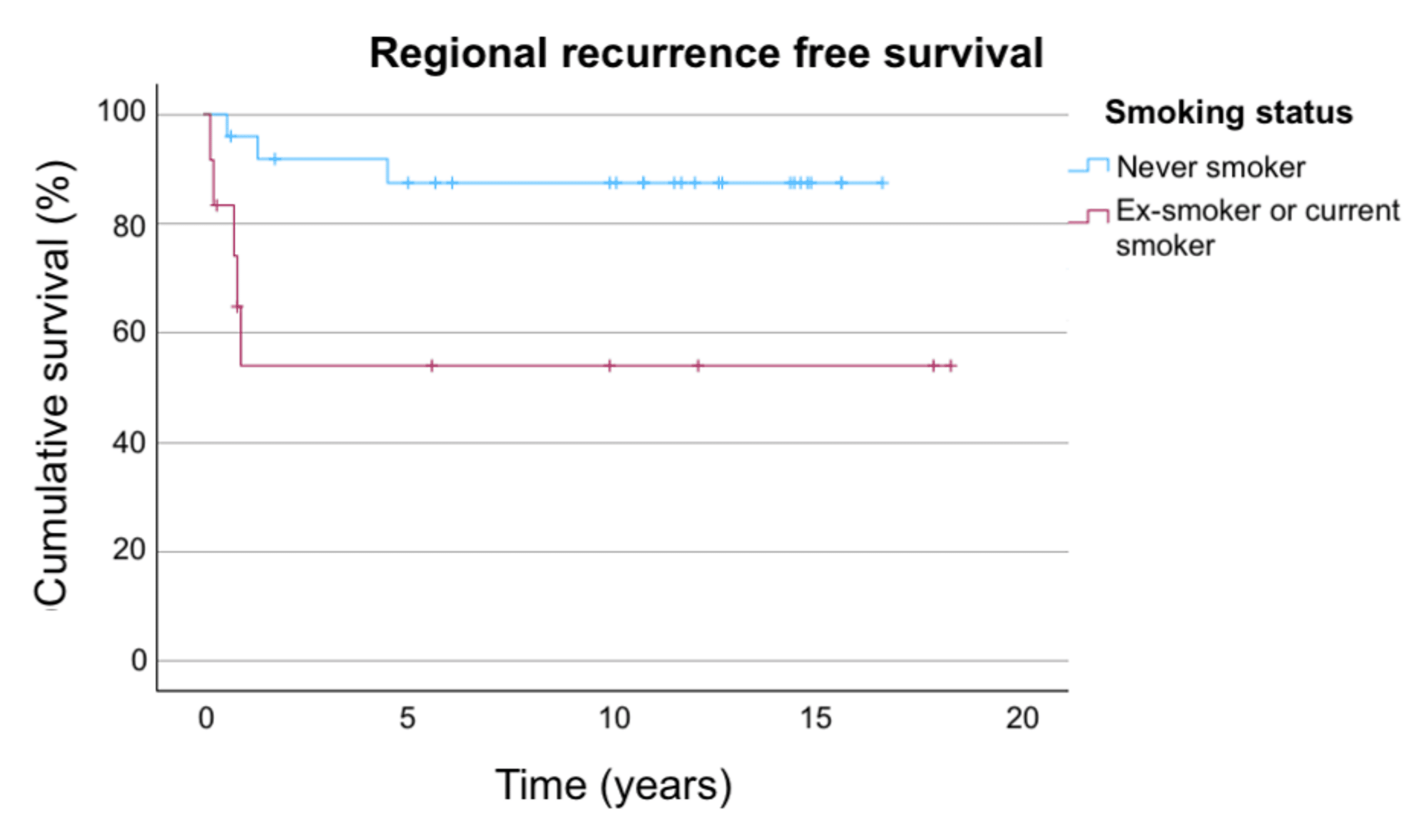
RRFS stratified by smoking status Figure 5 shows the RRFS stratified by smoking status. For never smokers, the three-year, five-year, and 10-year RRFS are 91.8%, 87.5%, and 87.5%, respectively. For ex-smokers or current smokers, the three-year, five-year, and 10-year RRFS are all 54%. RRFS: regional recurrence-free survival

The RRFS in the overall population, stratified by stage and smoking status, are shown in Figures 3-5.

The DSS was 88.6% at three years and at five years, and 82.3% at 10 years. Stage II oral cavity cancer was associated with numerically worse DSS but did not reach a statistically significant difference (HR: 4.11, 95% CI: 0.792-21.28, p=0.093). The other tested factors did not significantly impact the DSS, and they are listed in Table 4.

The DFS was 73% at three years, 62.2% at five years, and 56.8% at 10 years. However, a considerable proportion of deaths were related to intercurrent illness and not related to OCSCC. The DSS would be a better reflection of the overall survival in relation to the underlying cancer than DFS in the study population.

The OS was 78.4% at three years, 75.7% at five years, and 64.9% at 10 years. Smoking status and gender are factors that statistically significantly affect the outcome, with female patients having an HR of 0.382 (95% CI: 0.158-0.925, p=0.033) and smoking with an HR of 2.585 (95% CI: 1.066-6.271, p=0.036). Pathological node status was another significant prognostic factor, with higher nodal staging correlating with worse OS (HR: 1.732, 95% CI: 1.088-2.756, p=0.021); the main difference was driven by the pN2 group (HR: 8.68, 95% CI: 2.14-35.10, p=0.002). Other tested factors did not significantly impact the OS, which are listed in Table 4.

Local control

During the period of follow-up, 10 (26.3%) patients developed local recurrence, of whom six (60%) were salvaged by surgery. For the four patients who were not salvaged by surgery, three patients declined surgery because of the risks involved and one patient developed distant metastasis. The median time to local recurrence was 31 months (range: 2-134 months).

Stage I and stage II showed a numerical difference in local recurrence salvage rate, with salvage rate being 100% and 42.9% in stage I and stage II oral cavity cancer, respectively. However, this did not reach statistical significance (p=0.200 by Fisher’s exact test). There was no significant difference in salvage rate between HDR and LDR brachytherapy (p=0.524 by Fisher’s exact test).

The local control and salvage rate stratified by stage and type of brachytherapy (HDR or LDR) is presented in Table 5.

**Table 5:** Local control Table 5 shows the local recurrence rate and salvage rate by stage of disease. In each stage, the local recurrence rate and salvage rate are further analyzed with reference to the mode of brachytherapy used (HDR or LDR). HDR: high dose rate, LDR: low dose rate

		Overall	HDR	LDR
cT1N0	Local recurrence	3/21 (14.3%)	3/11 (27.3%)	0/10 (0%)
	Salvage rate	3/3 (100%)	3/3 (100%)	N/A
cT2N0	Local recurrence	7/17 (41.2%)	2/9 (22.2%)	5/8 (62.5%)
	Salvage rate	3/7 (42.9%)	1/2 (50%)	2/5 (40%)

Regional control

A total of eight (21.1%) patients developed regional recurrence, and three of them (37.5% who had regional recurrence) were salvaged by neck dissection. For the five patients who were not salvaged by surgery, three patients declined surgery because of the risks involved, one patient had inoperable regional recurrence, and one patient developed distant metastasis. The median time to regional recurrence was 9.5 months (range: 2-54 months).

Among the patients who had regional recurrence, six out of eight patients (75%) underwent initial elective neck node dissection following brachytherapy as initial treatment, and most (five out of six (83.3%)) had an initial pathologically node-negative state.

The regional recurrence salvage rate is not significantly different between stage I and stage II oral cavity cancer (p=1.00 by Fisher’s exact test) or brachytherapy by HDR or LDR (p=1.00 by Fisher’s exact test). The regional control and salvage rate stratified by stage and type of brachytherapy (HDR or LDR) is presented in Table 6.

**Table 6:** Regional control Table 6 shows the regional recurrence rate and salvage rate by stage of disease. In each stage, the regional recurrence rate and salvage rate are further analyzed with reference to the mode of brachytherapy used (HDR or LDR). HDR: high dose rate, LDR: low dose rate

		Overall	HDR	LDR
cT1N0	Regional recurrence	3/21 (14.3%)	2/11 (18.2%)	1/10 (10%)
	Salvage rate	1/3 (33.3%)	0/2 (0%)	1/2 (50%)
cT2N0	Regional recurrence	5/17 (29.4%)	3/9 (33.3%)	2/8 (25%)
	Salvage rate	2/5 (40%)	2/3 (66.7%)	0/2 (0%)

The regional recurrence rate for patients who underwent elective neck dissection after brachytherapy to tongue was numerically lower than those who did not undergo elective neck dissection, with a regional recurrence rate of 18.2% and 40%, respectively. This difference did not reach a statistical difference (p=0.279 by Fisher’s exact test).

Complications

For treatment-related complications at all grades, three (7.9%) patients developed persistent benign tongue ulcer, and four (10.5%) patients developed osteoradionecrosis of the jaw. There were no grade 3 or higher complications; all patients could be managed conservatively and did not require surgical intervention.

All patients with persistent benign tongue ulcer underwent biopsy to exclude local recurrence. The median time to ulcer onset was 41 months (range: 8-103 months). Smoking status was found to be statistically significantly associated with the occurrence of benign tongue ulcer (smoker versus non-smoker: 25% versus 0%, relative risk: 14.3, p=0.028 by Fisher’s exact test with Haldane correction). Cancer stage, mode of brachytherapy, and tumor site were not associated with the development of persistent tongue ulcer.

The median time to onset of osteoradionecrosis of jaw was 29 months (range: 13-96 months). No significant predictive factor was identified.

## Discussion

Local control

The five-year LRFS of patients who were treated by brachytherapy, with stage I and II combined, was 76.6% in the current study. This number was comparable to previous studies published since 2000, which evaluated the use of brachytherapy for early-stage OCSCC, with their five-year local control ranging from 65% to 100% [12-15].

There were two late recurrences that occurred at 121 and 134 months; given the long interval of more than 10 years from the diagnosis of primary tumor, these might represent second primary instead of local recurrence of the initial tumor. Given the challenges in differentiating a second primary from a late recurrence and that late local recurrences beyond five years after treatment have been reported [16,17], all tumors that were diagnosed in the treated side were counted as a local recurrent event so as to avoid underestimating treatment failure.

When stratified by stage, the five-year LRFS of patients treated by brachytherapy in the current study was 84.4% for stage I disease and 66.2% for stage II disease. In univariate analysis, patients with stage I OCSCC demonstrated numerically better outcomes than those with stage II disease, but the comparison did not reach statistical significance, possibly limited by the relatively small sample size. This was consistent with findings from previous studies, where a higher T stage was reported to have a significant correlation with local failure [15,17].

Previous studies reported the five-year local control rates for oral cavity cancer treated by surgery to be in the range of 81%-85% for stage I tumors and 77%-85% for stage II tumors [5]. Compared to the five-year LRFS of 84.4% and 66.2% for stage I and stage II disease, respectively, in this study, brachytherapy yielded a numerical comparable local control rate as surgery for stage I OCSCC, whereas the rate observed for stage II disease appeared lower than the outcomes reported for surgery. Based on these findings, the use of interstitial brachytherapy for early OCSCC may be more suitable for stage I disease.

Previous research evaluating stage I to II OCSCC demonstrated that patients who were treated primarily by surgery had a lower incidence of local recurrence compared to those receiving brachytherapy, with rates of 6% for surgery, and 17% and 35% for LDR and HDR brachytherapy, respectively [6]. While the local control for stage I OCSCC appeared numerically comparable to historical data for surgery, it should be highlighted that surgery remains the preferred treatment modality for OCSCC due to its superior oncological outcome. Interstitial brachytherapy represents an alternative that yields acceptable results in scenarios where surgical intervention is not feasible or when organ preservation is preferred.

The mode of brachytherapy, whether by HDR or LDR, did not significantly affect local control. HDR brachytherapy has the advantage of allowing the manipulation of source position and dwell time for optimization of dose distribution [3] and reducing radiation exposure to medical personnel due to its remote afterloading nature [18]. HDR brachytherapy has been more widely used in the recent decades. The recommended dose for HDR brachytherapy is 4 Gy for 11-12 fractions or 5-6 Gy for 9-10 fractions as per the Groupe Européen de Curiethérapie-European Society for Radiotherapy and Oncology (GEC-ESTRO) [19], 45-54 Gy in 10-12 fractions as per the Indian Brachytherapy Society [20], or 45-60 Gy at 3-6 Gy per fraction as per the National Comprehensive Cancer Network (NCCN) [1].

Early studies demonstrated that HDR brachytherapy resulted in less favorable outcomes compared to LDR for OCSCC. Lau et al. reported a lower local control rate and a tendency toward higher complication rates with HDR brachytherapy in node-negative squamous cell carcinoma of the tongue [21]. However, the radiotherapy regimen used in the study was 6.5 Gy for seven fractions (administered twice daily with a minimum interval time of six hours), which was calculated using the linear quadratic formula with an identical effect as conventional LDR prescription dose of 60 Gy. This dose fractionation is not a commonly used fractionation as recommended by the GEC-ESTRO, Indian Brachytherapy Society, or NCCN. The suboptimal oncological outcomes observed might be attributed to the prescribed dose rather than the mode of brachytherapy itself [21]. A more recent meta-analysis reported similar outcomes for HDR and LDR regarding local control, overall survival, and high-grade treatment-related complications [22]. The results of the current study aligned with these findings.

Upon local recurrence, salvage surgery was possible in six out of 10 (60%) of the relapsed patients in this study. Among those with local recurrence after initial stage I disease, the salvage rate was 100%, whereas it was 42.9% for patients with initial stage II disease. Similar findings were also reported in literature, where patients of higher T staging were less likely to be successfully salvaged by surgery [23].

Regional control

The optimal management of the neck in patients with early OCSCC undergoing interstitial brachytherapy remains a subject of debate. Various approaches have been described in the literature, including a watch-and-wait surveillance protocol, elective external beam radiotherapy to the neck, and elective neck dissection.

In patients with OCSCC who exhibited no clinical evidence of nodal metastasis, occult neck nodal metastases were identified in 25%-50% of cases [24]. The risk of occult neck nodal metastasis increases with a greater depth of tumor invasion, with a marked increase in the incidence of neck nodal metastasis from 5.6% with a depth of invasion of 3 mm to 16.9% with a depth of invasion of 4 mm [25]. In this study that included patients who had clinical stage I and II disease without evidence of nodal involvement, 27.4% of the patients were found to have neck nodal metastasis from elective neck dissection, which was consistent with the reported rate of occult neck nodal metastasis from literature. Pathological node status was a significant prognostic factor that impacted the OS, with a higher nodal staging correlating with worse OS (HR: 1.732, 95% CI: 1.088-2.756, p=0.021); the main difference was driven by the pN2 group (HR: 8.68, 95% CI: 2.14-35.10, p=0.002).

In this study, most patients (33 patients, corresponding to 86.8% of the patient population) underwent elective neck node dissection after brachytherapy. Some previous studies recommended a watch-and-wait surveillance strategy with close follow-up [24,26]. However, if a watch-and-wait surveillance strategy is adopted and neck nodal recurrence is found, successful salvage was only reported in around 50% of the patients [24]. More recent research showed that elective neck dissection in clinical node-negative T1-T2 OCSCC improved survival [25], and elective neck nodal dissection is strongly recommended for OCSCC with tumor depth > 3 mm [1]. Moreover, the regional recurrence rate for patients who underwent elective neck node dissection (18.2%) was numerically lower than that for patients who did not undergo elective neck node dissection (40%) in this study. Therefore, an elective regional nodal clearance would be considered a more prudent strategy following a radical treatment to the primary tumor, instead of a watch-and-wait strategy.

Elective neck node dissection was sequenced after interstitial brachytherapy to the primary in this study, as the recovery time following a neck dissection is often longer than that following interstitial brachytherapy to the primary, and that it would be preferable to sequence the treatment to the primary tumor first in a clinically uninvolved neck.

Importantly, our analysis on RRFS revealed that a shorter interval between the last brachytherapy fraction and the date of neck dissection was associated with numerically superior regional control. Although this association did not reach statistical significance, it might be advisable to consider an elective neck dissection promptly following the completion of interstitial brachytherapy to the primary site, to ensure timely management of occult regional disease.

Complications

Following interstitial brachytherapy for OCSCC, the incidence of soft tissue necrosis was reported to be 2%-16% in literature, whereas the incidence of osteoradionecrosis was reported in 1.1%-20% [5]. The incidence of significant complications necessitating surgical intervention was reported to be 2%-10% [24]. In the current study, soft tissue ulceration and osteoradionecrosis of the jaw was present in three (7.9%) and four (10.5%) patients, respectively, consistent with the previously reported incidence ranges.

Previous studies indicated that a higher tumor stage was associated with an increased incidence of complications [21,24]. In particular, a higher rate of significant complications was reported among patients with cT2 disease and tumor larger than 3 cm compared to cT1 disease, with a rate of severe soft tissue or bone complications reaching 24.4% in patients with tumor larger than 3 cm compared to 0.2% in patients with smaller tumors [24]. However, this association was not observed in the present study. This discrepancy might be attributed to the relatively smaller size of tumor in the patients in our study, which ranged from 0.3 cm to 3.0 cm. Since all patients in our study had tumor size measuring 3 cm or less, the correlation of tumor stage and incidence of complications might not be evident. Incidence of complications was comparable between patients treated with HDR or LDR brachytherapy, consistent with findings previously reported in the literature [12].

Notably, patients with current or past smoking history were more prone to the development of benign tongue ulcer. This would highlight the importance of smoking cessation in the treatment of OCSCC.

Limitations

The relatively small sample size of this study of 38 patients might limit the statistical power in the outcome analysis, particularly for the subgroup analyses and evaluation of prognostic variables, which would remain largely exploratory in nature. Due to the retrospective nature of this study, some information was not fully available, such as detailed grading of side effects, structured quality of life measures, and comprehensive assessment of functional outcome. The lack of a comparative group receiving upfront surgery for OCSCC in this study limited a direct comparison between surgery and brachytherapy regarding the outcomes and side effects. Consequently, results from previous studies on upfront surgery for OCSCC were referenced to evaluate the potential differences between surgery and brachytherapy.

The presence of these limitations underscores the importance of future research. Future prospectives studies may be considered with surgery and brachytherapy as comparative groups to evaluate the potential differences in treatment outcomes between the two modalities, including quality of life measures. This would yield high-quality evidence to enhance clinical decision-making for patients with early-stage OCSCC.

## Conclusions

Interstitial brachytherapy for early OCSCC yielded acceptable long-term tumor control and safety. Patients with stage I disease achieved numerically better local control and higher salvage rate after local relapse compared to those with stage II disease. Incorporating elective neck dissection following interstitial brachytherapy might offer additional improvements in oncological outcomes. While radical surgery continues to be the standard of care for OCSCC, interstitial brachytherapy remains an effective and safe alternative for selected patients with early-stage disease.
